# Long-term sickness absence among young and middle-aged workers in Norway: the impact of a population-level intervention

**DOI:** 10.1186/s12889-020-09205-3

**Published:** 2020-07-24

**Authors:** Therese N. Hanvold, Petter Kristensen, Karina Corbett, Rachel L. Hasting, Ingrid S. Mehlum

**Affiliations:** 1 Department of Occupational Health Surveillance, National Institute of Occupational Health, POB 5330 Majorstuen, N-0304 Oslo, Norway; 2 Department of Occupational Medicine and Epidemiology, National Institute of Occupational Health, Oslo, Norway; 3 Department of Mental Health and Suicide, Norwegian Institute of Public Health, Oslo, Norway; 4 Department of Community Medicine and Global Health, Institute of Health and Society, University of Oslo, Oslo, Norway

**Keywords:** Evaluation, Workplace, Sick leave, IA agreement

## Abstract

**Background:**

The study objective was to evaluate the impact of a population-level intervention (the IA Agreement) on the of one-year risk for long-term sickness absence spells (LSAS) among young and middle aged workers in Norway.

**Methods:**

Using an observational design, we conducted a quasi-experimental study to analyse registry data on individual LSAS for all employed individuals in 2000 (*n* = 298,690) and 2005 (*n* = 352,618), born in Norway between 1976 and 1967. The intervention of interest was the tripartite agreement for a more inclusive working life (the IA Agreement). We estimated difference in pre-post differences (DID) in LSAS between individuals working in IA companies with the intervention and companies without, in 2000 and 2005. We used logistic regression models and present odds ratios (DID OR) with accompanying 95% CI. We stratified analyses by sex, industry and company size.

**Results:**

We found no significant change in the overall risk of long-term sickness absence spells after implementing the intervention among young and middle aged workers. Stratified by sex, the intervention resulted in a slight decrease in LSAS risk among female workers (DID OR 0.93 (0.91–0.96)) while the intervention showed no impact among male workers (DID OR 1.01 (0.97–1.06)). We found that companies signing the IA Agreement were large (≥50 employees) and often within the manufacturing and health and social sectors. In large manufacturing companies, we found a reduction in LSAS, among workers both in companies with and without the intervention, resulting in no statistically significant impact of the IA intervention. In large health and social companies, we found an increase in LSAS among workers both in companies with and without the intervention. The increase was smaller among the workers in companies offering the IA intervention compared with workers in companies without, resulting in a positive impact of the IA intervention in the health and social industry. This impact was statistically significant only among female workers.

**Conclusions:**

The results indicate that the impact of the IA Agreement on the risk of long-term sickness absence spells varies considerably depending on sex and industry. These findings suggest that reducing LSAS may warrant industry-specific interventions.

## Background

Sickness absence (SA) remains a significant problem globally with a financial and health burden for societies and individuals. In Norway, the level of SA is considered relatively high compared to neighbouring countries [1]. Consequently, reducing SA is an important political objective and initiatives with this aim have received significant attention. One such initiative was introduced in 2001, where the employer organizations, employee organizations, and the Government signed the Tripartite Agreement for a More Inclusive Working Life (the IA Agreement). The IA Agreement’s three main goals were to reduce SA, secure recruitment of people with disabilities and vulnerable groups into the labour market, and prolong working life [2]. The IA Agreement can be considered a population-level “intervention”, where companies voluntarily signed an IA Agreement with the Norwegian Labour and Welfare Administration (NAV). Signing an IA Agreement granted access to consulting services and subsidies to assist the companies’ work on reducing sickness absence and increasing work participation. This “intervention” is a large societal investment constituting a major cost, as the budget for one year of these services has been estimated to be 38 million euros [3]. The Agreement has been renewed five times, most recently in December 2018 for the period 2019–2022. Its latest renewal entails an expansion meaning that the IA Agreement comprises all of Norway’s companies and employees. It also entails a clearer focus on reducing SA and decreasing the withdrawal from work life. The agreement also states that the efforts should target the long-term and recurring sickness absence spells. Despite its latest expansion and its high cost, there is still a lack of effect-focused studies on its impact [4]. There have been previous non-scientific evaluations of the intervention, but these have seldom taken into account the heterogeneity between companies with and without the IA Agreement, such as company size and industry [3, 5, 6]. Moreover, the few scientific studies assessing the IA Agreement’s effectiveness have given contradictory results. Two studies showed no impact on SA [7, 8], while more recent studies, using causal inference methods, found positive impacts with a higher probability of returning to work [9] and a lower likelihood of receiving a full disability pension [10]. These studies are, however, done on selected samples, such as participants on work rehabilitation [9] and older employees aged 50–61 [10], highlighting the need for studies on other samples/cohorts.

In the present observational study, we have access to data on all individuals born in Norway between 1967 and 1976, their individual-level SA, and the IA status of the company they work in pre and post intervention period (2000 and 2005). These data provide an opportunity to examine the IA Agreement’s impact on achieving the goal to reduce SA in a young/middle aged population of workers (age span 24–38 years during follow-up). This is an especially important group to examine, as SA early in working life is associated with later SA and thus lower work participation [11]. We have chosen to examine long-term sickness absence spells as these constitute the largest part of sick leave in Norway [12], increase the risk of withdrawal from work life and have a large impact on social expenditure. By linking individual-level data from several registers with company-level data on the IA status and using a quasi-experimental design, our aim was to evaluate the impact of this population-level intervention on long-term sickness absence spells (LSAS). The assignment of companies and workers into the IA agreement or not was outside the control of the study and was registered retrospectively. We performed analyses stratified by sex, company size and industry, as previous evaluations showed that the distribution of company size and industry varies by IA status [3, 13], and that SA varies substantially between sex and industry [14]. Distinguishing between possible different impacts of the intervention by industry and company size may assist future prioritizations of certain groups.

## Methods

The IA Agreement in Norway, first introduced in 2001, is the population-level intervention of interest in this study. Using an observational design, we conducted a quasi-experimental study following recent guidelines on evaluating population health interventions [15]. The intervention group (individuals working in companies with an IA Agreement) was compared with the control group (individuals working in companies without an IA Agreement) regarding their pre-post differences in LSAS in the study periods 2000 and 2005.

### Data source

The data material comes from a cohort consisting of all individuals born in Norway between 1967 and 1976 (*n* = 626,928). They have been identified through the national identification number in the Medical Birth Registry of Norway allowing for data linkage across several national registries. Background characteristics and data on SA and industry were available from Statistics Norway’s events database on employment and welfare, FD-Trygd [16]. National registries are updated either on an annual basis, or are event-history databases, in which case we have the precise dates of the events. In addition, we have obtained annual data from the registries of NAV on the companies’ size and the companies’ annual IA status. It was possible to link this information to the individuals’ employment in a company with or without the IA Agreement.

### Design and population

**Fig. 1:**
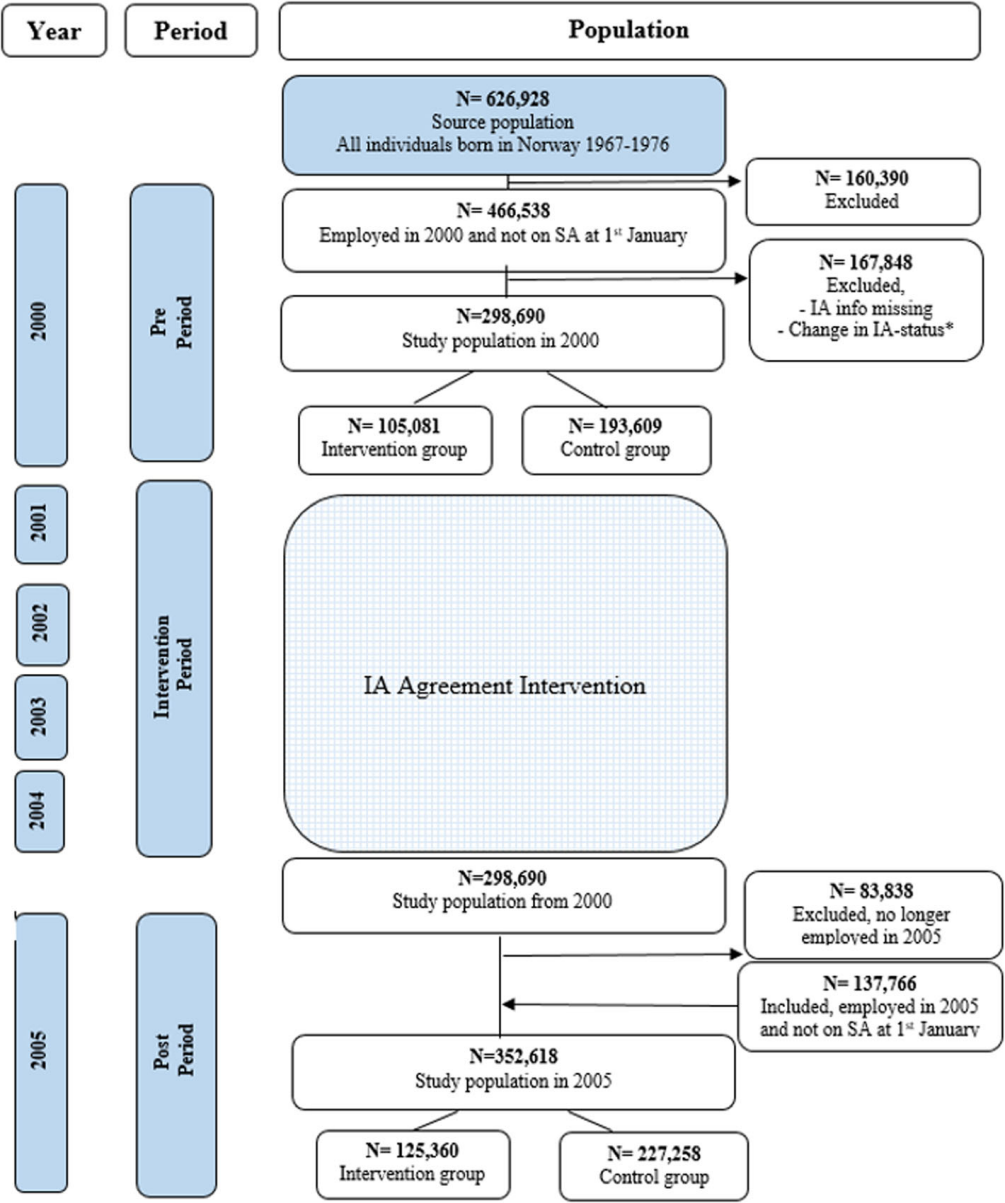
Flowchart, showing study population and intervention. *Excluded if they had missing information of IA status or was working in a company that had signed the IA Agreement after 2003 or a company had left the IA Agreement prior to 2005. For those present in both years were excluded if they had changed from intervention to control group or vice versa

Figure 1 illustrates the design and a flowchart of the source population (*n* = 626,928). The study population consisted of those employed and not on SA at the beginning of the year (2000: *n* = 298,690, 2005: *n* = 352,618). We compared the annual risk of LSAS in the pre period (year 2000) and the post period (year 2005) between those who worked in a company with an IA Agreement (intervention group) and those who worked in companies without an IA Agreement (control group). The intervention period ranged from January 2001, to December 2004. Only those employed and not on SA on 1st January 2000 were included in the pre period (*N* = 466,538). Individuals were excluded if they; (i) worked in a company with missing information on IA status, (ii) were working in a company that had signed the Agreement after 2003, or (iii) had left the Agreement prior to 2005. Of the initial population, 36% (*N* = 167,848) were excluded according to these criteria. The study population in 2000 was comprised of 298,690 individuals, of which 105,081 were in the intervention group and 193,609 were in the control group. New individuals were included in 2005 if they were employed and not on SA on 1st January 2005; after applying the above exclusion criteria, this resulted in an additional 137,766 individuals and a total study population in 2005 of 352,618 individuals (125,360 in the intervention group and 227,258 in the control group).

### Intervention (the IA agreement)

Our independent variable measured whether the participants worked in a company that had signed the IA Agreement in the period 2001–2003. We considered signing the Agreement in 2004 or later as too late in terms of evaluating the impact on LSAS in 2005. Signing an IA Agreement gives the companies access to funding of both individual interventions, to retain employees at work during illness, and group-based preventative interventions. The interventions include opportunities for tighter and earlier follow-up of workers with LSAS, increased use of graded sickness certification, and access to subsidies for work adjustments. Furthermore, IA companies have access to “NAV working life centres” that assist companies in their strategic work related to the goals of the IA Agreement [1]. Thus, signing the IA Agreement can be seen as a proxy for initiating specific interventions to prevent LSAS among the company’s employees. The NAV data enabled classification of the treatment and control groups by identifying employees working in companies with and without an IA Agreement on an annual basis. The IA Agreement status (intervention assignment) was therefore not at the discretion of the researcher.

### Outcome (long-term sickness absence spells)

LSAS data were obtained from the event database FD-Trygd, which records all physician-diagnosed SA spells lasting > 16 calendar days (LSAS). Employees in Norwegian companies receive full salary from the employer during certified sickness absence. NAV reimburses the employer for absences lasting > 16 calendar days, and these absence spells are registered in the database; therefore, registration is considered to be complete for employees.

We obtained individual records on long-term sickness absence spells (> 16 days) for the periods January 1st to December 31st of 2000 and 2005. We estimated the risk of having one or more long-term sickness absence spells during the one-year period among those who were at risk at the beginning of the year, for 2000 and 2005, respectively (cumulative incidence).

There are many ways to measure SA, the most common being workdays on sick leave as a proportion of expected workdays [6]. This measure is used when presenting the national statistics on SA. However, in an epidemiological framework, the proper quantification depends strongly on the aim and study sample. Five valuable measures have however been proposed in a review on how to measure SA, and cumulative incidence is one of these [17]. We chose to use cumulative incidence because the same measure of SA has been used in earlier scientific papers evaluating the IA Agreement’s impact on sickness absence [7, 8], thus enabling easier comparisons of results. Our choice was also based on a belief that LSAS could capture changes resulting from the IA interventions, which aim to reduce long lasting and recurring sickness absence spells, and may lead to shorter (< 16 days) and fewer SA spells.

### Covariates

Data on sex and year of birth were obtained from the Medical Birth Registry of Norway.

Information on industry was obtained from Statistics Norway’s FD-Trygd database on an annual basis and was coded according to the Standard Industrial Classification 2002 [16]. This classification has a hierarchical, top-down structure that begins with general characteristics and narrows down to more specific job areas. The first two digits of the code represent the major industries to which a company belongs [18]. In this study we have identified 13 industries; 1 Agriculture/forestry A/B), 2 Mining/quarrying (C), 3 Manufacturing (D), 4 Electricity, gas and water supply (E), 5 Construction (F), 6 Wholesale and retail (G), 7 Hotel and restaurant work (H), 8 Transport and storage (I), 9 Financial and real estate (J/K), 10 Public administration (L), 11 Education (M), 12 Health and social work (N), 13 Other community and social work (P/Q).

Data on company size were obtained from the registers of NAV on an annual basis and divided in three different groups: (i) small companies (0–10 employees), (ii) medium-sized companies (11–49 employees) and (iii) large companies (> 50 employees).

### Statistics

We estimated the one-year risk of one or more long-term sickness absence spells during the pre-intervention period (year 2000) and post-intervention period (year 2005). No information was available on the specific interventions used in the companies; thus, our analysis evaluates the total impact of working in a company that has signed the Agreement and possibly started offering workplace interventions to reduce employees’ LSAS. We used the difference-in-difference (DID) method, which can account for fixed unobserved individual differences. The counterfactual method inherent in DID allows us to evaluate the LSAS for the control group IF they hypothetically had received the treatment/intervention and is thus a reflection of the change in the intervention group due to the intervention.

We calculated pre-post differences between the intervention and the control group. The impact of the IA Agreement on LSAS was estimated as the difference in pre-post differences (differences between 2005 and 2000) between the IA and non-IA groups, using logistic regression models [19]. The Odds Ratios (DID OR) with accompanying 95% confidence intervals (CI) are presented and the statistical significance level was set at a *p*-value < 0.05. An OR value < 1.0 indicates that the additive difference in LSAS risk between 2005 and 2000 (risk_2005_ – risk_2000_) in the intervention group is lower than the corresponding figure in the control group. This is referred to as a positive impact of the intervention on LSAS.

The DID method has a strong common trend assumption, namely that the intervention and control groups would have followed the same trajectory had the intervention not taken place. This was checked through comparing LSAS with common trend graphs in a period prior to the intervention (1998–2000) and the assumption was considered satisfied (see Supplementary Figure 1).

We also estimated the marginal effect, where the percentage point (PP) indicates a positive impact of the intervention if the number is negative. A negative impact of the intervention on LSAS is shown by an OR value > 1.0, and indicates that the additive difference in LSAS risk in 2005 and 2000 (risk_2005_ – risk_2000_) in the intervention group is higher than the corresponding figure in the control group. In the marginal effect estimates, this is indicated as a positive number.

Based on earlier studies evaluating the IA Agreement, we know that it was unlikely that the intervention and control group would be similar in all manners. We therefore stratified the analyses by sex, industry and company size. To ensure statistical power in the adjusted analyses, only industries with > 2000 employees in each group (control and intervention) for both years (2000 and 2005) were included. This led to subgroup analyses in manufacturing, construction, wholesale/retail, transport/storage, financial/real estate, public administration, education and health/social work. STATA/SE 14.0 Software was used for analyses (STATA Corporation, College Station, Texas, U.S.A). The study was approved by the Regional Committees for Medical and Health Research Ethics (REK).

## Results

Table 1 shows that the mean age of employees in companies with and without an IA Agreement was similar, however, the two groups differed when it came to important variables such as sex, company size and industry. A higher proportion of employees in companies with IA Agreement were female (58%) and working in large companies (70%). They were most commonly employed in health/social work (35%), manufacturing (15%) and education (14%). Employees in the control group, on the other hand, were most commonly employed in wholesale/retail (26%), financial and real estate (18%) and manufacturing (14%). They were more evenly distributed between small (28%), medium-sized (37%), and large companies (34%). The two groups also differed when it came to LSAS, as the employees in companies signing the IA Agreement had a 3 percentage point (PP) higher one-year risk of LSAS prior to the intervention compared with employees in control companies (16.9 and 13.9%, respectively). See Table 1 for more study population characteristics.

**Table 1:** Descriptive statistics for employees in companies with an intervention (IA) and employees in companies without an intervention, Controls (Non-IA)

	2000				2005			
	Intervention *N* = 105,081 (35.2)		Control *N* = 193,609 (64.8)		Intervention *N* = 125,360 (35.5)		Control *N* = 227,258 (64.5)	
	N	%	N	%	N	%	N	%
**Age**								
Mean (SD)	28.9 (2.8)		28.5 (2.8)		33.7 (2.8)		33.6 (2.8)	
**Sex**								
Female	60,959	58	85,691	44	72,318	58	93,777	44
**Company size**								
Small (0–10 employees)	4572	4	53,915	28	5761	5	68,449	32
Medium (11–49 employees)	26,384	25	70,529	37	33,122	26	84,858	39
Large (≥50 employees)	73,000	70	65,152	34	86,474	69	63,985	29
**Industry**								
Agriculture/forestry (A,B)	277	0.3	3635	2	414	0.3	3344	2
Mining/quarrying (C)	1195	1	2054	1	1964	2	3328	2
Manufacturing^a^ (D)	15,873	15	27,439	14	16,142	13	28,517	13
Electricity, gas and water supply (E)	588	0.6	815	0.4	1195	1.0	2482	1
Construction^a^ (F)	4493	4	16,267	8	4866	4	23,282	10
Wholesale and retail^a^ (G)	4761	4	50,969	26	4617	4	50,723	22
Hotel and restaurant work (H)	1668	2	10,306	5	1106	1	6560	3
Transport and storage^a^ (I)	6602	6	13,933	7	7156	6	16,898	8
Financial and real estate^a^ (J/K)	6068	6	35,051	18	7305	6	41,016	18
Public administration^a^ (L)	9915	9	2276	1	13,232	11	6488	3
Education^a^ (M)	15,096	14	5153	3	21,100	17	8028	4
Health and social work^a^ (N)	36,449	35	16,203	8	46,752	35	25,776	11
Other community and social work (P/Q)	2066	2	9421	5	2380	2	10,261	5
**Sickness absence**								
LSAS (risk of long-term sickness absence spells > 16 days duration)	17,804	17	26,921	14	22,034	18	32,708	14

^a^Industrial sectors selected in analyses

**Fig. 2:**
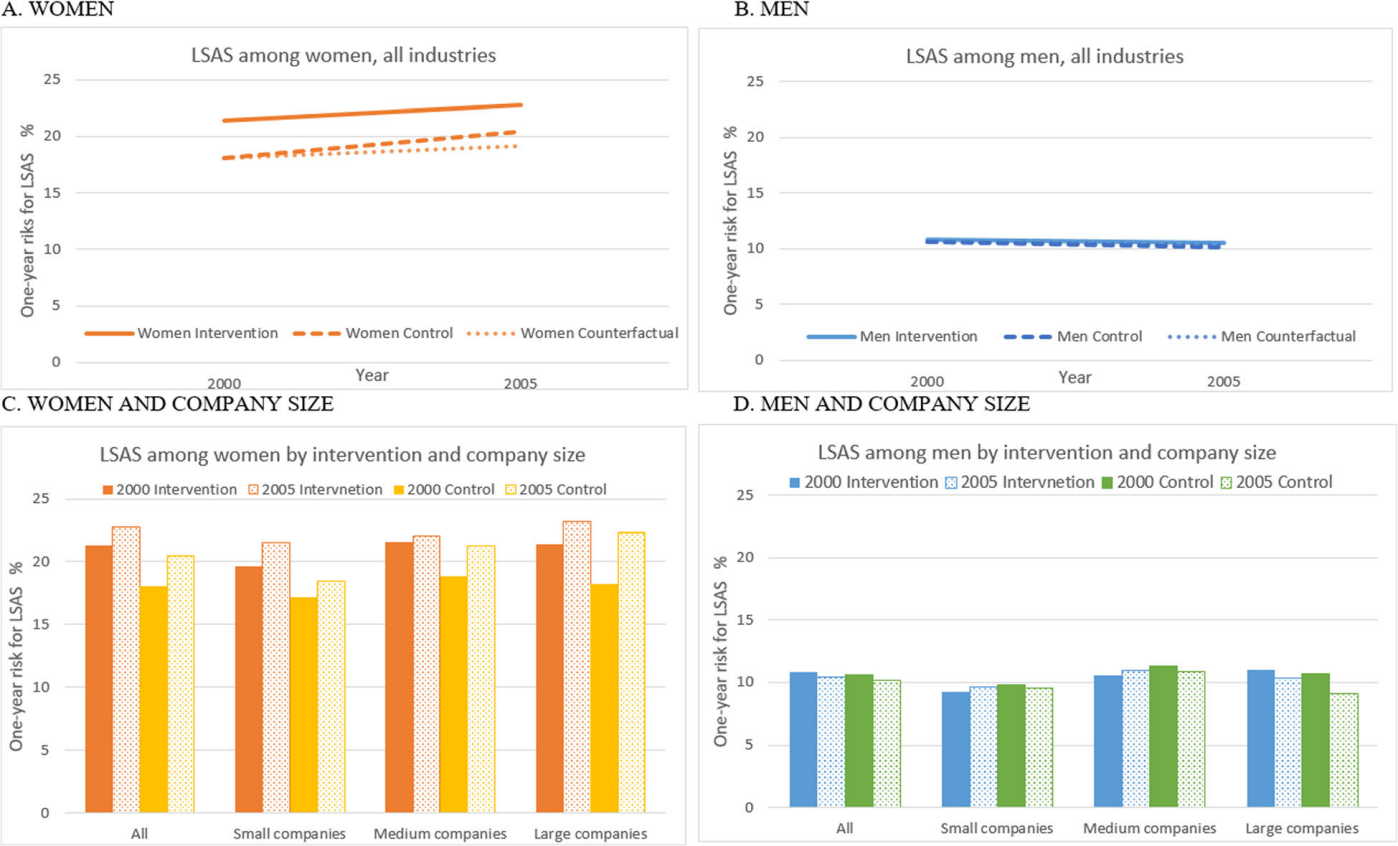
Risk of LSAS in intervention and control group by sex and company size

Figure 2a and b show the risk of LSAS in the control and treatment groups for women and men, respectively, and for the period pre and post IA Agreement. From 2000 to 2005, we found an overall increase in LSAS among women in both the intervention (21.4 to 22.8%) and control group (18.0 to 20.4%). Men showed no changes in LSAS in either the intervention (10.8 to 10.5%) or the control group (10.6 to 10.1%). Among women, accounting for the differences in LSAS at baseline, and despite the increase in LSAS for both intervention and control group, a significant positive impact of having an IA Agreement was found (OR 0.93, CI 0.91–0.96). Among men, however, when taking into account the differences in the LSAS at baseline, no significant impact of the IA Agreement was found (OR 1.01, CI 0.97–1.06). Figure 2c and d show the risk of LSAS in the control and treatment group for women and men, respectively, by company size. As SA increases with age, we conducted supplementary analyses where age was adjusted for in the overall results to account for possible effects of being four years older. In these supplementary analyses, the risk of LSAS is more similar in the pre and post period and the impact of the IA Agreement remained the same, but slightly reduced. Figure 2c suggests that there is a positive impact of the IA Agreement for women in large and medium-sized companies; even though the risk of LSAS increases in both the control and intervention groups, it increases more in the control group than the intervention group. For men, a negative impact of the IA Agreement was found in large companies; even though the risk of LSAS decreases in both the control and intervention groups, it decreases more in the control group than the intervention group.

Our evaluation of the impact of the Norwegian Agreement for a More Inclusive Working Life shows no significant overall impact on the risk of LSAS after implementing the intervention. In this period, from 2000 to 2005, we found an overall increase in risk of LSAS in both the intervention (16.9 to 17.6%) and control group (13.9 to 14.4%). The results, however, show that the impact of the implemented intervention varied by industry and company size. In Fig. 3.1 and 3.2, we present the estimated impact of the IA Agreement for large companies in eight selected industries, for men and women separately. This is a choice based on the available sample size; see Supplementary Table 1 and 2 for estimates for all company sizes. For women in large companies within the health and social work sector, the financial/real estate and public administration sectors we found a positive impact of the IA Agreement. For men in large companies, we found a positive impact of the IA Agreement within the health and social work sector, the wholesale/retail and transport sector. No impact was found within large companies in manufacturing, construction or education.

**Fig. 3:**
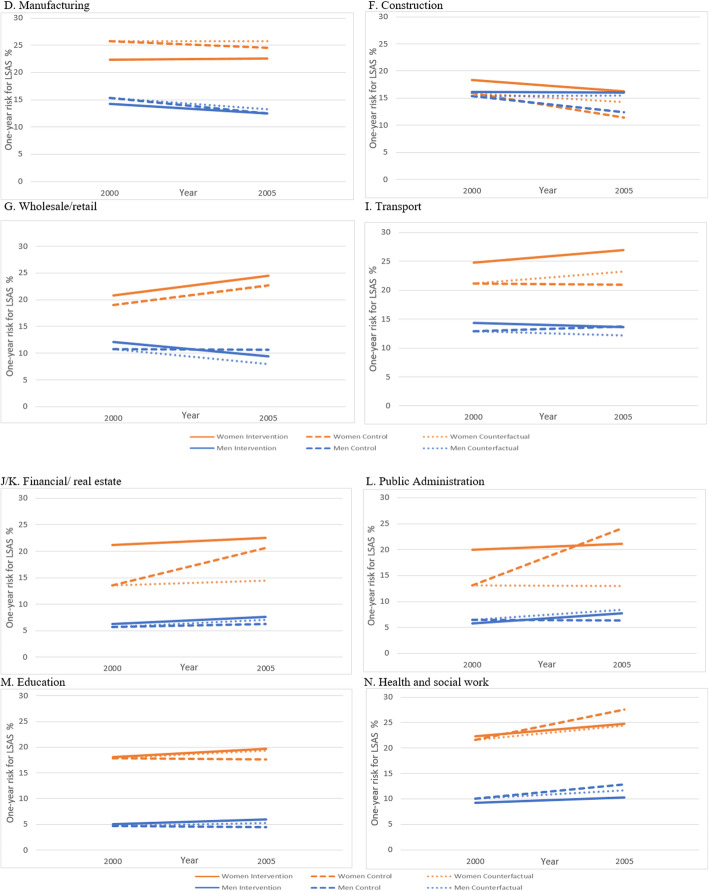
.1 and 3.2 Risk of LSAS in intervention and control groups in large companies (≥50 employees), by selected industries and sex

In Table 2, we present the impact of the IA Agreement adjusted for company size and industry. For women the Agreement had an overall significant positive impact, decreasing the one-year risk of LSAS by 1.3 PP. For the different industries, our results indicate a positive impact of the IA Agreement among female workers in large companies in the public administration sector (11.1 PP decrease in LSAS risk) and female employees working in the health and social work sector (ranging from 2.3 PP for employees in medium companies to 3.2 PP for employees in large companies). For male workers, our overall results show no impact on the one-year risk of LSAS. Among men, the only industry that showed a significant positive impact of the IA Agreement was among workers in large wholesale and retail companies (2.6 PP decrease in LSAS risk). There was a significant negative impact in large companies in the construction and public administration sectors (3.1 PP and 2.1 PP increase in LSAS risk, respectively).

**Table 2:** Adjusted difference-in-difference estimates of the impact of the IA agreement on the risk of long-term sickness absence spells, by sex, industry and company size

	WOMEN			MEN		
	DID OR	(95%CI)	Effect^c^ PP	DID OR	(95%CI)	Effect^c^ PP
**All**^a^	0.93	(0.91–0.96)	−1.1	1.01	(0.97–1.06)	0.1
All^b^	0.92	(0.89–0.95)	− 1.3	1.02	(0.96–1.08)	0.2
**Manufacturing (D)**	1.01	(0.90–1.13)	0.2	1.02	(0.93–1.12)	0.2
Small (0–10 employees)	0.57	(0.18–1.84)	−8.4	0.32	(0.04–2.44)	− 10.9
Medium (11–49 employees)	0.90	(0.78–1.04)	− 1.6	1.04	(0.75–1.43)	0.4
Large (≥50 employees)	1.07	(0.93–1.24)	1.3	1.07	(1.00–1.16)	0.8
**Construction (F)**	1.81	(1.43–2.29)	6.4	1.16	(0.92–1.46)	1.8
Small (0–10 employees)	0.95	(0.39–2.31)	−0.5	2.20	(1.01–4.79)	8.7
Medium (11–49 employees)	2.83	(1.32–6.08)	10.7	1.04	(0.85–1.27)	0.5
Large (≥50 employees)	1.25	(0.51–3.06)	2.9	1.28	(1.09–1.50)	3.1
**Wholesale and retail (G)**	0.96	(0.86–1.08)	− 0.6	0.89	(0.76–1.06)	−1.0
Small (0–10 employees)	1.02	(0.77–1.33)	0.2	1.49	(0.93–2-40)	3.4
Medium (11–49 employees)	0.86	(0.75–0.97)	−2.5	0.98	(0.81–1.19)	− 0.1
Large (≥50 employees)	0.99	(0.68–1.44)	− 0.1	0.76	(0.62–0.93)	−2.6
**Transport and storage (I)**	1.12	(0.98–1.27)	1.9	0.96	(0.86–1.06	− 0.5
Small (0–10 employees)	0.72	(0.32–1.62)	−4.8	0.21	(0.45–1.02)	−15.6
Medium (11–49 employees)	1.11	(0.81–1.54)	1.7	1.49	(1.19–1.86)	4.7
Large (≥50 employees)	1.13	(0.99–1.28)	2.2	0.88	(0.61–1.12)	−1.5
**Financial/real estate (J/K)**	0.85	(0.72–0.98)	−2.3	1.08	(0.94–1.24)	0.4
Small (0–10 employees)	2.24	(1.36–3.69)	10.7	1.06	(0.32–3.55)	0.3
Medium (11–49 employees)	1.09	(1.01–1.17)	1.2	0.93	(0.64–1.35)	−0.4
Large (≥50 employees)	0.65	(0.60–0.71)	−6.2	1.15	(0.84–1.59)	0.8
**Public administration (L)**	0.73	(0.65–0.83)	−4.8	1.12	(0.84–1.49)	0.7
Small (0–10 employees)	1.62	(0.62–4.21)	6.6	2.63	(1.19–5.84)	6.9
Medium (11–49 employees)	0.90	(0.57–1.44)	−1.5	1.31	(0.55–3.08)	2.0
Large (≥50 employees)	0.50	(0.34–0.72)	−11.1^a^	1.40	(1.05–1.84)	2.1
**Education (M)**	1.03	(0.89–1.89)	0.5	0.91	(0.73–1.14)	−0.8
Small (0–10 employees)	1.44	(0.94–2.23)	5.2	1.57	(0.52–4.68)	3.0
Medium (11–49 employees)	0.93	(0.71–1.20)	−1.2	0.81	(0.52–1.24)	−1.3
Large (≥50 employees)	1.13	(0.94–1.35)	1.8	1.16	(0.84–1.60)	0.8
**Health and social work (N)**	0.98	(0.93–1.04)	−0.3	0.92	(0.76–1.11)	− 0.8
Small (0–10 employees)	0.97	(0.84–1.10)	−0.5	1.00	(0.62–1.60)	0.0
Medium (11–49 employees)	0.88	(0.81–0.95)	−2.3	0.96	(0.63–1.46)	−0.3
Large (≥50 employees)	0.84	(0.79–0.89)	−3.2	0.88	(0.66–1.18)	− 1.1

*DID* Difference-in-difference, *OR* Odds Ratios, *CI* Confidence Interval, *PP* Percentage Point (%)

All analyses adjusted for age. ^a^Unadjusted. ^b^Adjusted for company size and industry ^c^ Average Marginal Effect

## Discussion

We found no significant impact overall, of implementing the IA Agreement on the risk of long-term sickness absence spells. When stratifying by sex, there was an overall positive impact of the IA Agreement among female workers, whilst no effect was found among male workers. Companies signing the Agreement were more likely to be large (≥50 employees) and were more often within the manufacturing and health and social work sectors. In large manufacturing companies, there was a statistically significant reduction in the risk of LSAS among both male and female workers after the implementation of the IA Agreement. As this was found in both intervention and control companies, it indicates no impact of the IA intervention. In large health and social companies there was, in contrast, an increase in the risk of LSAS after the introduction of the IA Agreement. The increase was lower in the intervention group compared to the control group, resulting in a positive impact of the actual IA intervention. This pattern was mainly evident among female workers in large health and social companies. In sum, the results indicate that the impact of the IA Agreement on risk of LSAS varied considerably depending on sex, industry, and company size.

### Methodological considerations

One of the strengths of this study is the use of statistical analyses that take into account the difference in LSAS pre and post intervention. Difference-in-difference analysis is a causal inference method that can be applied to counter selection bias and confounding. The large study population also made it possible to stratify by sex, industry and company size. This stratification, in combination with the DID analyses, could therefore reduce the bias and confounding that may result from the significant differences in the distribution of company size, industry and sex between the intervention and control groups. This difference in the distribution of the IA Agreement by industry and company size was, however, a challenge in some strata where groups were small, leading to less robust estimates. In addition, self-selection bias may be an issue, as we observed that the employees in companies signing the IA Agreement had a higher risk of LSAS prior to the intervention, compared with employees in control companies. This might challenge the key assumption of exogeneity in DID, which posits that the selection into the intervention (the IA Agreement) should not be predicted by the outcome (LSAS) prior to the intervention. However, there is no evidence that individuals choose where they work based on the company’s IA status, and the companies do not only base their choice of signing the IA Agreement on prior sickness absence level [20]. It is also worth mentioning that the NAV Working Life Centres had a recruitment campaign which mainly targeted companies with high SA, and we cannot rule out that this may have influenced risk of bias.

Difference-in-difference analysis is often used to counter selection bias between the intervention and control group, including for repeated cross-sectional data where the two samples are not the same in the pre and post period [21]. The DID method can therefore account for changes within the groups over time, as long as the change is the same in both the intervention and control groups. Age is an example of this in our study, as the groups both age by 4 years (see Table 1). This change should therefore not influence our results, as the effect of age is assumed to be equal for both groups. This is supported by the supplementary analyses we conducted where the risk of LSAS was higher but the direction of impact of the IA Agreement remained the same, but slightly reduced (see supplementary Figure 2).

One of the many challenges in evaluating the IA Agreement’s impact on LSAS was that we did not have data on exactly when the IA Agreement was signed by a specific company (only yearly data). We did not have data on when they started introducing the different preventive measures, either. Other studies have shown, however, that most companies signing the Agreement increased their effort to lower SA following the start of the IA Agreement in 2001, regardless of the date they formally signed the Agreement [5]. Even so, the specific preventive actions or activities they may offer is not available in our data, preventing us from evaluating the possible differential impact of activities on SA. To address this, we have used company size as a proxy measure as this can indicate differences in the means or resources they have available to use in implementing the IA activities, applying for grants and so on. Larger companies may have more resources available to make use of and benefit from the possibilities in the IA Agreement more than small companies.

Sickness absence is multi-factorial and influenced not only by the individual’s health status and work environment, but also by the regulations in the welfare system. A challenge often encountered when evaluating population-level interventions is other “interventions” outside the scope of the evaluation that impact the outcome. During the intervention period in our study, a sick leave reform was introduced. The reform was implemented in 2004, and a key element was the activity requirement. It required an individual to engage in work-related activity as early as possible (at the latest within eight weeks) in order to be entitled to sick pay. The only exception was when medical reasons clearly prevent it. SA figures from Statistics Norway show a marked decline in sick leave in 2004 and 2005 that coincides with the sick leave reform. It is most likely that the 2004 reform has an effect on LSAS in our study, but this effect is assumed to be equal in both groups as the change in regulations was implemented nationally and applied to all companies, regardless of the companies’ IA status.

Another limitation of this study is the focus on LSAS alone as the outcome, as the IA Agreement incorporates three goals that might influence each other. Reducing SA is only one of the goals. The two other goals are to secure recruitment of people with disabilities and vulnerable groups into the labour market and to prolong working life. These three goals may affect each other, as a company that increases the recruitment of people with disabilities and prolongs working life for older workers, might also experience increased SA due to this. It is therefore possible that we may present an underestimation of the impact of the intervention on LSAS in our study for companies with a high goal attainment on inclusion of disabled workers, but this is unknown. It is therefore important to have in mind that our results are only evaluating one of the goals of the IA Agreement.

### Our results in light of other findings

Our finding that the impact of the IA Agreement on LSAS varied considerably depending on sex and industry contributes to limited literature on the impacts of this population based intervention. Evaluations of the IA Agreement have been published in some reports without peer review [5, 6, 22, 23], and have indicated a positive impact of the IA Agreement in manufacturing, as this sector shows a decrease in LSAS after implementation of the intervention. Earlier reports have also indicated a negative impact of the intervention in the health and social work sector, as LSAS increases in this sector in the same period [5]. Our results were therefore a bit surprising as we found the opposite; there was no impact of the IA Agreement in manufacturing, and a positive impact in health and social work. These contradicting results can be explained by the fact that we use an analytical method that takes into account the intervention and control group differences, both before the IA Agreement was implemented and 4 years after. This explanation is strengthened by the fact that we get similar findings (decrease in LSAS for employees in manufacturing companies and increase in LSAS for employees in health and social work companies) when we use the same statistical methods as in the reports, without the use of DID method.

It is also important to bear in mind that how sickness absence is estimated, can lead to contrasting results when interpreting the impact of the IA Agreement. We evaluated the impact on the change in one-year risk of long-term sickness absence spells. We cannot rule out the possibility that a different result may have been obtained if a different measure had been used such as the mean duration of sickness absence spells, the annual number of spells or the use of graded sickness absence spells. However, other scientific papers on the IA Agreement use the same measure of SA as in this study, enabling easier comparisons of results. A study from 2011, on the impact of the IA Agreement on sickness absence [7] shows similar results as in this study, namely no impact in the overall sample. However, in contrast, we find that the impact varies by industry. This may partly be explained by the considerably smaller sample in the 2011 study, which impedes stratification by industry. In their analysis, they also used office workers as a reference, and did not include information on company size or take into account the baseline difference in sickness absence. Another study by Midtsundstad et al. from 2014 showed, on the other hand, a positive impact of the intervention on overall sickness absence and a varying effect by industry [8]. They used the same DID method as in our study and found a positive impact on sickness absence among IA companies in the public administration sector. This is partially in line with our findings; however, in our study, the positive impact in the public administration sector was only found in women and varied according to company size. They also did not find positive impacts of the intervention for manufacturing, construction and transport; although the overall sickness absence decreased in manufacturing, it was not due to the intervention, which is also similar to our findings. This can indicate that the decrease in sickness absence may be related to other factors than the IA Agreement, for example company characteristics or the focus on sickness absence and work environment in the manufacturing sector as a whole, resulting in an impact for all companies and not only those signing the IA Agreement. We also found a positive impact among female health and social workers in medium and large companies, which was not found in the other study [8]. A potential reason for this discrepancy in results may be that they focused on older workers (aged 50+) whilst our study included younger and middle-aged workers (aged 25–34). Beyond these two studies, there is little scientific knowledge on the impact of the IA Agreement on sickness absence that also takes into account the possible differential impacts by industry. Other scientifically based evaluations of the IA Agreement have been carried out, largely reporting a positive impact, however, they have evaluated other outcomes, such as disability benefits [10] and return to work after rehabilitation [9], and are therefore not comparable to our study.

### Implications

The present study strengthens the evidence that the impact of the IA Agreement on the risk of long-term sickness absence spells varies greatly between industries and the size of the companies. Very few clear implications can be given based on this study, as we do not have data on the preventive measures used in different sectors. The varying impact by industry may however, imply that the IA Agreements measures (such as focusing on close follow up of those on sick leave, adjust work tasks to enable the employee to work even when sick) may suit the needs of some industries and not others. For example, in some industries the possibilities to adapt the work tasks are more limited than in others, such as manual workers in manufacturing or construction compared with office workers in public administration. These measures may therefore not be suitable for all industries.

It is, also evident that the impact varies greatly depending on the size of the company, which may imply that larger companies have more resources available to make use of and benefit from the IA Agreement’s activities compared to smaller companies. Based on this it is evident that there is a need for a greater focus on industry-related exposures and the possibilities for preventive measures addressing the specific challenges in each industry. Previous studies have found that 23–28% of long-term sickness absence is attributable to work-related exposures [24, 25]. This indicates that interventions targeting the work environment can be considered an important method for decreasing sickness absence. According to the surveillance of work environment for the working population in Norway [14], health and social workers have challenges in terms of high emotional demands, role conflicts, job strain, unwanted sexual attention, violence and threats, working nights, neck bending, and awkward lifting. In contrast, manufacturing workers have more exposure to noise, vibrations, awkward lifting, squatting/kneeling, downsizing, and job insecurity. This may indicate that reducing sickness absence in these two sectors would warrant different preventive strategies and actions.

## Conclusion

This study provides knowledge on the differential impacts of the IA Agreement on the risk of long-term sickness absence spells by sex, industry and company size. The results indicate that reducing LSAS may warrant industry-specific interventions. The data available, however, gives us no possibility to interpret which of the IA activities are effective. Future research should therefore obtain data that can more precisely reflect IA activity at the company level and focus on evaluating specific preventive measures, as some measures may have more impact on LSAS than others.

## Supplementary information

**Additional file 1: Figure S1.** Description: LSAS in intervention and control group in the pre intervention period 1998–2000. **Figure S2.** Description: Risk of LSAS in intervention and control group by sex, adjusted for age.

**Additional file 2: Table S1.** Description: Distribution and crude LSAS risks for female employees according to year, intervention status, industrial sector, and company size. ** Table S2.** Description: Distribution and crude LSAS risks for male employees according to year, intervention status, industrial sector, and company size.

## Data Availability

The data are not publically available as restrictions apply to the availability of the data. The permissions was approved from REC (ref.no. S-06028a), FD-trygd and NAV. Contact corresponding author for more information on data availability.

## Abbreviations

CI: Confidence Interval
DID: Difference- in- Difference
IA: Inclusive working life Agreement
LSAS: Long-term sickness absence spells
NAV: The Norwegian Labour and Welfare Administration
OR: Odds Ratio
PP: Percentage Point
REK: The Regional Committees for Medical and Health Research Ethics
SA: Sickness Absence
