# Supplementary material for: Long-term sickness absence among young and middle-aged workers in Norway: the impact of a population-level intervention
Source: BMC Public Health. 2020 Jul 24;20:1157. doi: 10.1186/s12889-020-09205-3 (PMC7379790; doi:10.1186/s12889-020-09205-3)

Figure 1S LSAS in intervention and control group in the pre intervention period 1998-2000

Figure 2S Risk of LSAS in intervention and control group by sex, adjusted for age


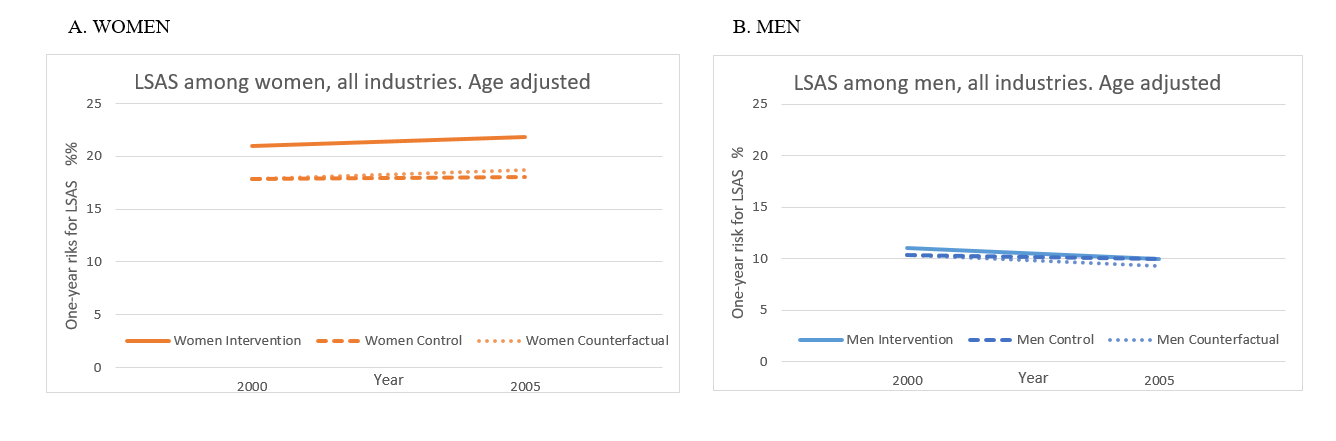

Supplement: Supplementary file 1 — Additional file 1: Figure S1. Description: LSAS in intervention and control group in the pre intervention period 1998–2000. Figure S2. Description: Risk of LSAS in intervention and control group by sex, adjusted for age. [file 12889_2020_9205_MOESM1_ESM.docx]
