# Supplementary material for: Long-term sickness absence among young and middle-aged workers in Norway: the impact of a population-level intervention
Source: BMC Public Health. 2020 Jul 24;20:1157. doi: 10.1186/s12889-020-09205-3 (PMC7379790; doi:10.1186/s12889-020-09205-3)
Supplement: Supplementary file 2 — Additional file 2: Table S1. Description: Distribution and crude LSAS risks for female employees according to year, intervention status, industrial sector, and company size. Table S2. Description: Distribution and crude LSAS risks for male employees according to year, intervention status, industrial sector, and company size. [file 12889_2020_9205_MOESM2_ESM.docx]

Supplementary Table 1. Distribution and crude LSAS risks for female employees according to year, intervention status, industrial sector, and company size

|  |  |  | 2000 | | | | |  | 2005 | | | | |
| --- | --- | --- | --- | --- | --- | --- | --- | --- | --- | --- | --- | --- | --- |
|  |  |  |  | |  |  | |  |  | |  |  | |
|  |  |  | Intervention  N=60,959 | |  | Control  N=85,691 | |  | Intervention  N=72,318 | |  | Control  N= 93,777 | |
|  |  |  | N | LSAS % |  | N | LSAS % |  | N | LSAS % |  | N | LSAS % |
|  | |  |  |  |  |  |  |  |  |  |  |  |  |
| **Industrial sector** | | **Company size** |  |  |  |  |  |  |  |  |  |  |  |
|  | Manufacturing |  | 3,878 | 22.6 |  | 7,423 | 22.4 |  | 3,953 | 22.4 |  | 7,184 | 22.0 |
|  |  | Small (0-10 employees) | 61 | 31.1 |  | 1,132 | 16.5 |  | 33 | 24.2 |  | 1,245 | 19.8 |
|  |  | Medium (11-49 employees) | 445 | 22.9 |  | 2,407 | 20.2 |  | 526 | 20.9 |  | 2,426 | 19.9 |
|  |  | Large (≥50 employees) | 3,349 | 22.4 |  | 3,822 | 25.7 |  | 3,394 | 22.6 |  | 3,416 | 24.5 |
|  | Construction |  | 497 | 17.5 |  | 1,070 | 17.3 |  | 576 | 15.3 |  | 2,502 | 8.7 |
|  |  | Small (0-10 employees) | 18 | 22.2 |  | 400 | 16.2 |  | 51 | 11.8 |  | 1,027 | 8.6 |
|  |  | Medium (11-49 employees) | 118 | 13.5 |  | 387 | 19.9 |  | 187 | 14.4 |  | 1,059 | 8.4 |
|  |  | Large (≥50 employees) | 355 | 18.3 |  | 264 | 15.9 |  | 338 | 16.3 |  | 341 | 11.4 |
|  | Wholesale and retail |  | 1,833 | 19.4 |  | 24,579 | 18.3 |  | 1,746 | 21.8 |  | 23,036 | 21.3 |
|  |  | Small (0-10 employees) | 274 | 16.8 |  | 9,921 | 16.7 |  | 350 | 20.0 |  | 10,336 | 19.6 |
|  |  | Medium (11-49 employees) | 844 | 19.2 |  | 10,691 | 19.8 |  | 726 | 20.2 |  | 9,210 | 23.5 |
|  |  | Large (≥50 employees) | 705 | 20.8 |  | 3,408 | 19.0 |  | 670 | 24.5 |  | 2,916 | 22.7 |
|  | Transport and storage |  | 2,749 | 23.8 |  | 3,852 | 19.6 |  | 2,715 | 26.7 |  | 4,178 | 19.7 |
|  |  | Small (0-10 employees) | 38 | 28.9 |  | 741 | 17.7 |  | 78 | 21.8 |  | 923 | 17.0 |
|  |  | Medium (11-49 employees) | 616 | 20.1 |  | 977 | 16.1 |  | 488 | 26.4 |  | 1,162 | 19.6 |
|  |  | Large (≥50 employees) | 2,037 | 24.8 |  | 2,065 | 21.2 |  | 2,149 | 26.9 |  | 2,003 | 21.0 |
|  | Financial and real estate |  | 3,151 | 19.8 |  | 16,049 | 13.9 |  | 3,465 | 22.4 |  | 16,517 | 18.2 |
|  |  | Small (0-10 employees) | 142 | 9.8 |  | 3,359 | 14.2 |  | 200 | 23.0 |  | 4,686 | 16.7 |
|  |  | Medium (11-49 employees) | 636 | 17.4 |  | 3,920 | 15.0 |  | 753 | 21.8 |  | 5,481 | 17.6 |
|  |  | Large (≥50 employees) | 2,345 | 21.2 |  | 8,470 | 13.5 |  | 2,512 | 22.5 |  | 5,964 | 20.6 |
|  | Public administration |  | 5,471 | 19.3 |  | 1,244 | 14.4 |  | 7,151 | 20.4 |  | 2,382 | 19.7 |
|  |  | Small (0-10 employees) | 300 | 13.7 |  | 227 | 15.8 |  | 416 | 19.9 |  | 297 | 15.5 |
|  |  | Medium (11-49 employees) | 1,340 | 18.5 |  | 375 | 15.7 |  | 1,630 | 18.4 |  | 683 | 16.9 |
|  |  | Large (≥50 employees) | 3,808 | 20.0 |  | 625 | 13.1 |  | 5,105 | 21.1 |  | 974 | 24.1 |
|  | Education |  | 10,366 | 18.9 |  | 3,480 | 18.1 |  | 13,899 | 19.8 |  | 4,800 | 18.5 |
|  |  | Small (0-10 employees) | 398 | 14.8 |  | 402 | 15.4 |  | 443 | 21.4 |  | 556 | 16.5 |
|  |  | Medium (11-49 employees) | 5,209 | 20.2 |  | 1,636 | 19.2 |  | 6,745 | 19.8 |  | 2,341 | 20.0 |
|  |  | Large (≥50 employees) | 4,605 | 18.1 |  | 1,394 | 17.8 |  | 6,711 | 19.7 |  | 1,756 | 17.6 |
|  | Health and social work |  | 29,885 | 22.8 |  | 13,675 | 21.7 |  | 35,574 | 24.7 |  | 21,223 | 23.8 |
|  |  | Small (0-10 employees) | 1,308 | 24.5 |  | 3,770 | 21.0 |  | 1,674 | 22.7 |  | 5,173 | 19.9 |
|  |  | Medium (11-49 employees) | 6,388 | 24.6 |  | 4,928 | 22.6 |  | 8,602 | 24.9 |  | 8,120 | 25.1 |
|  |  | Large (≥50 employees) | 21,876 | 22.3 |  | 4,746 | 21.6 |  | 25,298 | 24.8 |  | 5,230 | 27.6 |

LSAS: long-term sickness absence spells

Supplementary Table 2. Distribution and crude LSAS risks for male employees according to year, intervention status, industrial sector, and company size

|  |  | |  |  | 2000 | | | | |  | 2005 | | | | |
| --- | --- | --- | --- | --- | --- | --- | --- | --- | --- | --- | --- | --- | --- | --- | --- |
|  |  | |  |  |  | |  |  | |  |  | |  |  | |
|  |  | |  |  | Intervention  N=44,122 | |  | Control  N=107,918 | |  | Intervention  N=53,042 | |  | Control  N= 133,481 | |
|  |  | |  |  | N | LSAS % |  | N | LSAS % |  | N | LSAS % |  | N | LSAS % |
|  | |  | |  |  |  |  |  |  |  |  |  |  |  |  |
|  | | **Industrial sector** | | **Company size** |  |  |  |  |  |  |  |  |  |  |  |
|  |  | | Manufacturing |  | 11,995 | 14.3 |  | 20,016 | 14.3 |  | 12,189 | 12.5 |  | 21,333 | 12.3 |
|  |  | |  | Small (0-10 employees) | 163 | 17.2 |  | 3,197 | 11.8 |  | 131 | 5.3 |  | 4,294 | 10.1 |
|  |  | |  | Medium (11-49 employees) | 1,261 | 14.1 |  | 7,447 | 14.4 |  | 1,587 | 13.2 |  | 8,404 | 13.0 |
|  |  | |  | Large (≥50 employees) | 10,496 | 14.3 |  | 9,150 | 15.3 |  | 10,471 | 12.5 |  | 8,327 | 12.5 |
|  |  | | Construction |  | 3,996 | 15.7 |  | 15,197 | 14.7 |  | 4,290 | 15.5 |  | 20,780 | 12.8 |
|  |  | |  | Small (0-10 employees) | 160 | 10.0 |  | 5,865 | 13.6 |  | 189 | 17.5 |  | 8,078 | 12.0 |
|  |  | |  | Medium (11-49 employees) | 1,294 | 15.6 |  | 6,360 | 15.6 |  | 1,489 | 14.4 |  | 8,801 | 13.9 |
|  |  | |  | Large (≥50 employees) | 2,523 | 16.2 |  | 2,625 | 15.4 |  | 2,612 | 16.0 |  | 3,451 | 12.4 |
|  |  | | Wholesale and retail |  | 2,928 | 11.4 |  | 26,390 | 9.8 |  | 2,871 | 10.8 |  | 27,687 | 10.3 |
|  |  | |  | Small (0-10 employees) | 268 | 7.5 |  | 9,286 | 8.9 |  | 406 | 11.8 |  | 10,674 | 9.8 |
|  |  | |  | Medium (11-49 employees) | 1,505 | 11.6 |  | 11,553 | 10.3 |  | 1,300 | 11.7 |  | 11,628 | 10.5 |
|  |  | |  | Large ≥50 employees) | 1,129 | 12.1 |  | 5,042 | 10.7 |  | 1,165 | 9.4 |  | 4,918 | 10.6 |
|  |  | | Transport and storage |  | 3,853 | 13.7 |  | 10,081 | 12.4 |  | 4,441 | 13.9 |  | 12,720 | 13.1 |
|  |  | |  | Small (0-10 employees) | 68 | 17.6 |  | 2,882 | 10.9 |  | 62 | 4.8 |  | 3,435 | 12.0 |
|  |  | |  | Medium (11-49 employees) | 650 | 11.5 |  | 2,605 | 13.7 |  | 660 | 16.2 |  | 3,643 | 13.6 |
|  |  | |  | Large (≥50 employees) | 3,012 | 14.3 |  | 4,395 | 12.9 |  | 3,719 | 13.6 |  | 5,289 | 13.7 |
|  |  | | Financial and real estate |  | 2,917 | 6.7 |  | 19,002 | 5.6 |  | 3,840 | 7.8 |  | 24,499 | 6.1 |
|  |  | |  | Small (0-10 employees) | 173 | 5.2 |  | 3,879 | 5.2 |  | 230 | 6.5 |  | 7,071 | 6.1 |
|  |  | |  | Medium (11-49 employees) | 598 | 9.2 |  | 4,846 | 5.9 |  | 861 | 8.8 |  | 8,337 | 6.1 |
|  |  | |  | Large (≥50 employees) | 2,108 | 6,2 |  | 9,675 | 5.7 |  | 2,748 | 7.6 |  | 8,390 | 6.2 |
|  |  | | Public administration |  | 4,444 | 6.4 |  | 1,032 | 7.5 |  | 6,081 | 8.2 |  | 4,106 | 6.5 |
|  |  | |  | Small (0-10 employees) | 312 | 8.0 |  | 188 | 11.7 |  | 430 | 8.1 |  | 319 | 5.1 |
|  |  | |  | Medium (11-49 employees) | 1,055 | 7.9 |  | 206 | 6.6 |  | 1,421 | 9.4 |  | 553 | 6.1 |
|  |  | |  | Large (≥50 employees) | 3,070 | 5.8 |  | 524 | 6.5 |  | 4,230 | 7.7 |  | 1,988 | 6.3 |
|  |  | | Education |  | 4,730 | 5.7 |  | 1,673 | 5.5 |  | 7,201 | 6.4 |  | 3,228 | 5.6 |
|  |  | |  | Small (0-10 employees) | 155 | 7.1 |  | 195 | 9.7 |  | 217 | 6.9 |  | 419 | 6.4 |
|  |  | |  | Medium (11-49 employees) | 1,795 | 6.2 |  | 767 | 5.3 |  | 2,697 | 7.1 |  | 1,143 | 7.6 |
|  |  | |  | Large (≥50 employees) | 2,701 | 5.4 |  | 684 | 4.7 |  | 4,287 | 6.0 |  | 1,326 | 4.4 |
|  |  | | Health and social work |  | 6,564 | 9.4 |  | 2,528 | 9.3 |  | 8,178 | 10.7 |  | 4,553 | 11.5 |
|  |  | |  | Small (0-10 employees) | 183 | 9.8 |  | 499 | 8.8 |  | 213 | 11.7 |  | 850 | 10.8 |
|  |  | |  | Medium (11-49 employees) | 951 | 10.2 |  | 1,070 | 8.7 |  | 1,376 | 12.3 |  | 1,810 | 10.7 |
|  |  | |  | Large (≥50 employees) | 5,368 | 9.2 |  | 917 | 10.1 |  | 6,589 | 10.3 |  | 1,146 | 12.8 |

LSAS: Long-term sickness absence spells
